# Stakeholder expectations from the integration of chiropractic care into a rehabilitation setting: a qualitative study

**DOI:** 10.1186/s12906-018-2386-3

**Published:** 2018-12-04

**Authors:** Zacariah K. Shannon, Stacie A. Salsbury, Donna Gosselin, Robert D. Vining

**Affiliations:** 1 Palmer Center for Chiropractic Research, Palmer College of Chiropractic, 741 Brady Street, Davenport, IA 52803 USA; 2 Milford, NH, USA

**Keywords:** Chiropractic, Complementary therapies, Integrative medicine, Rehabilitation, Treatment outcome, Attitude of health personnel, Qualitative research, Hospitals, special, Musculoskeletal pain

## Abstract

**Background:**

Few studies have investigated patient and provider expectations of chiropractic care, particularly in multidisciplinary settings. This qualitative study explored stakeholder expectations of adding a chiropractor to the healthcare team at a rehabilitation specialty hospital.

**Methods:**

The research methodology was an organizational case study with an inpatient facility for persons recovering from complex neurological conditions serving as the setting. Sixty stakeholders, including patients, families, hospital staff, and administrators, were interviewed or participated in focus groups in June 2015. Semi-structured questions guided the interview sessions which were digitally audiorecorded and transcribed. Data were entered into a qualitative software program to conduct content analysis using an iterative approach to identify key themes.

**Results:**

Expectations for the chiropractic program were mostly positive with themes consistently reported across stakeholder groups. The central domain, *making progress*, encompassed the organizational mission to empower patients to reach hospital discharge and return to life in the community. Higher order goals, characterized as achieving *whole person healing*, encompassed patients’ quality of life, self-efficacy, and activities of daily living. Stakeholders expected the addition of chiropractic to help patients progress toward these goals by improving pain management and physical functioning. *Pain management* themes included pain intensity, medication use, and pain-related behaviors, while *functional improvement* themes included muscle tone, extremity function, and balance and mobility. In addition to these direct effects on clinical outcomes, stakeholders also expected indirect effects of chiropractic care on *healthcare integration*. This indirect effect was expected to increase patient participation in other providers’ treatments leading to improved care for the patient across the team and facility-level outcomes such as decreased length of stay.

**Conclusions:**

Stakeholders expected the addition of chiropractic care to a rehabilitation specialty hospital to benefit patients through pain management and functional improvements leading to whole person healing. They also expected chiropractic to benefit the healthcare team by facilitating other therapies in pursuit of the hospital mission, that is, moving patients towards discharge. Understanding stakeholder expectations may allow providers to align current expectations with what may be reasonable, in an effort to achieve appropriate clinical outcomes and patient and staff satisfaction.

## Background

Patient expectations of healthcare interventions, that is, their beliefs about “the services they think they are to receive” [1], are thought to play an important role in both clinical outcomes and patient satisfaction [2–4]. Patients with musculoskeletal (MSK) conditions expect improved outcomes from biomedical procedures, such as joint surgery, as well as complementary treatments, including acupuncture, massage, and spinal manipulation [5]. Patients also have expectations regarding how this healthcare is delivered [6, 7].

Studies of the expectations of complementary and alternative medicine (CAM) users have investigated therapies for chronic low back pain (LBP) [8–11]. Generally, patient expectations included pain relief and improvements in functional activities, muscle strength and flexibility, mood, and quality of life [9]. Unanticipated outcomes described by these CAM users were an increased sense of hope and well-being; improved relaxation, energy, or body awareness; better coping abilities and patient activation; and improvements in overall health and non-LBP-related conditions [8]. A reframing of expectations for pain relief, along with an increased ability to cope with pain, refined bodily awareness, and new acceptance of living with chronic LBP were reported by CAM users in longitudinal interviews [11].

While chiropractic patients were included in the preceeding studies, the expectations of chiropractic users are not well known and focus on patients seeking care in outpatient settings only. Sigrell’s studies of patients with sub-acute LBP in Sweden reported patient expectations of chiropractic management as accurate diagnosis, treatment and advice for the problem, and a positive outcome [12, 13]. Patients and chiropractors viewed a positive outcome as being ‘free of symptoms’ but just what these symptoms might be were not explicated [12, 13]. MacPherson and colleagues conducted a survey of treatment expectations with 544 chiropractic patients in the UK [14]. Respondents reported beneficial outcomes from chiropractic care for pain, mobility, flexibility, health maintenance, and prevention of future problems, as well as an understanding of their health problems, confidence in self-management, and return to work or activities [14]. No previous studies have explored patient expectations of chiropractic care among persons in inpatient settings, such as rehabilitation hospitals.

Further, the expectations that medical personnel have about chiropractic are not well-known, even for healthcare professionals who work with chiropractors in multidisciplinary facilities. A case study of chiropractic clinics in public sector healthcare facilities noted mixed expectations among medical providers, administrators and support staff, with favorable expectations for the treatment of back pain, particularly among providers with a familiarity with chiropractic care [15]. However, explicit outcome domains, such as pain or function, were not identified. Among medical colleagues at 9 private sector healthcare facilities, chiropractic care was viewed favorably for patients with MSK conditions, especially in the areas of pain management, functional limitations, and patient satisfaction [16].

Doctors of chiropractic (DC), patients, families, and medical providers might benefit from sharing mutual expectations of the outcomes of chiropractic care. Without such shared expectations among key stakeholders, patient progress might be hindered either directly, by not addressing patient or provider expectations, or indirectly, by leading to team dysfunction from ill-defined roles or by not meeting the overall goals of the healthcare team. The purpose of this qualitative study was to explore stakeholder expectations for the integration of a DC into the healthcare team at a rehabilitation specialty hospital.

## Methods

### Design overview and ethics considerations

We conducted a longitudinal case study using qualitative methods (interviews, focus groups) as part of a larger research project to evaluate the process and outcomes of adding a DC to the clinical team in a multidisciplinary rehabilitation hospital [17–19]. The Institutional Review Boards of Palmer College of Chiropractic and Crotched Mountain Foundation provided the research ethics approvals for this project. All participants signed a written informed consent. This report describes stakeholder outcome expectations expressed during baseline interviews conducted before the implementation of the chiropractic program. The methods for this case study are described in greater detail elsewhere [19].

### Setting

Crotched Mountain Specialty Hospital (CMSH), a 62-bed skilled nursing facility, served as the study setting. CMSH inpatient units focused on sub-acute, multidisciplinary rehabilitation for adult and pediatric patients with brain injury (traumatic or other type) and spinal cord injury. Adult inpatients, the focus on this research project, varied in health status substantially from ambulatory patients treated in community-based chiropractic settings [20]. For example, most CMSH patients (85%) used a wheelchair as their primary means of mobility and more than half required moderate to complete assistance in activities of daily living [17]. CMSH patients had varied musculoskeletal impairments including spine-related pain (54%), joint stiffness (50%), and extremity pain (46%), with 85% of patients reporting pain in the past 5 days and 63% describing that pain as moderate to severe in intensity [17]. Of a sample (*n* = 27) described in a companion study to this report, 63% of patients had an interdisciplinary care plan for pain management, 67% used non-pharmacological therapies to address pain, and 22% were on a scheduled program of pain medications [17].

### Participants and recruitment

Participants were adults age 18 years or older who were stakeholders in the mission of CMSH, including patients, families, hospital personnel, and community members. Exclusions to participation were an unwillingness to consent to an audiorecorded interview and, for patients, the presence of a health condition that prevented verbal communication during the interview. Hospital personnel who concurrently served on the research team recruited participants using role-specific brochures and personal invitations. Hospital staff recruited included medical physicians and nurse practitioners; nursing staff; physical, occupational and speech therapists and assistant staff; psychologists; therapeutic recreational staff; and administrative personnel. Therapy and nursing staff were offered a $25 gift card to compensate for extended work hours from study involvement and snacks during the focus groups which were scheduled during work breaks. No other incentives were provided to participants.

### Data collection

Investigators from the chiropractic research center (SAS, RDV) conducted all interviews and focus group sessions during an intensive, week-long, site visit to CMSH designed for this purpose. Most interview sessions were conducted by a single interviewer (SAS). Individual interviews were completed with patients, family members, community stakeholders, administrators, and members of the medical staff. Professional staff and assistive personnel from the therapy department and registered nurses and licensed assistants from the nursing department completed role-specific focus groups. Interviews were completed in June 2015, with the DC interviewed at a later time following hire.

Investigators used a semi-structured interview guide with questions tailored to participant role. During the introductory portion of the interviews, the interviewers identified ourselves as a registered nurse (SAS) or doctor of chiropractic (RDV) who worked at the chiropractic research center so our professional backgrounds were apparent to participants. Participants were asked to speak freely about the topic of chiropractic care so that the research team could understand better how chiropractic might fit within the larger work of this healthcare organization. Participants were encouraged to voice both positive experiences or negative concerns about chiropractic and, when such personal reflections were accounted (especially in focus groups), the interviewers prompted the other participants to recount similar or differing experiences. Overall, participants seemed to have few difficulties in sharing their stories about chiropractic. Many people described how either they themselves or a family member had received chiropractic manipulations in the past, often attributing specific clinical outcomes to that chiropractic care. Further, several longer-term staff offered similar accounts of a past experience with a local chiropractor, which helped the researchers understand why some clinicians expressed caution about the new program.

During the interview sessions, several questions generated extended discussion about patient and provider expectations for the chiropractic program, including: 1) How do you think a chiropractor might help your/your patients’ current condition; 2) How will the inclusion of chiropractic services affect the delivery of patient care at this facility; and 3) If successful, what impact will the chiropractic program have on patient care? Interviews were audiorecorded digitally for transcription by a professional service (Way With Words, New York, NY, USA).

### Data analysis

Verbatim transcriptions of audio-recordings were completed, quality checked, and entered into a qualitative data analysis program (NVivo©, Version 9.2, QSR International Pty Ltd., Victoria, Australia). As most interviews were conducted during a single site visit, data analysis followed the completion of all interviews and focus groups. As we have described elsewhere [19], four coders, including three research assistants overseen by a study investigator (SAS), conducted the qualitative content analysis using an inductive approach [21]. Team members read the transcripts independently, coded the transcripts, and made written memos or annotations on their analysis process. The research team met weekly as a group to refine the codebook, deliberate divergences in the coding process, identify emerging insights, and to discuss such issues as researcher or professional identity to serve as a means of identifying, if not bracketing, potential sources of biases in our analyses [22]. Themes were categorized into domains with shared attributes. All transcripts were coded over multiple rounds of iterative coding. For this paper, we present themes related to expected outcomes from the chiropractic program only; other findings from the qualitative interviews are presented elsewhere [19].

## Results

**Fig. 1:**
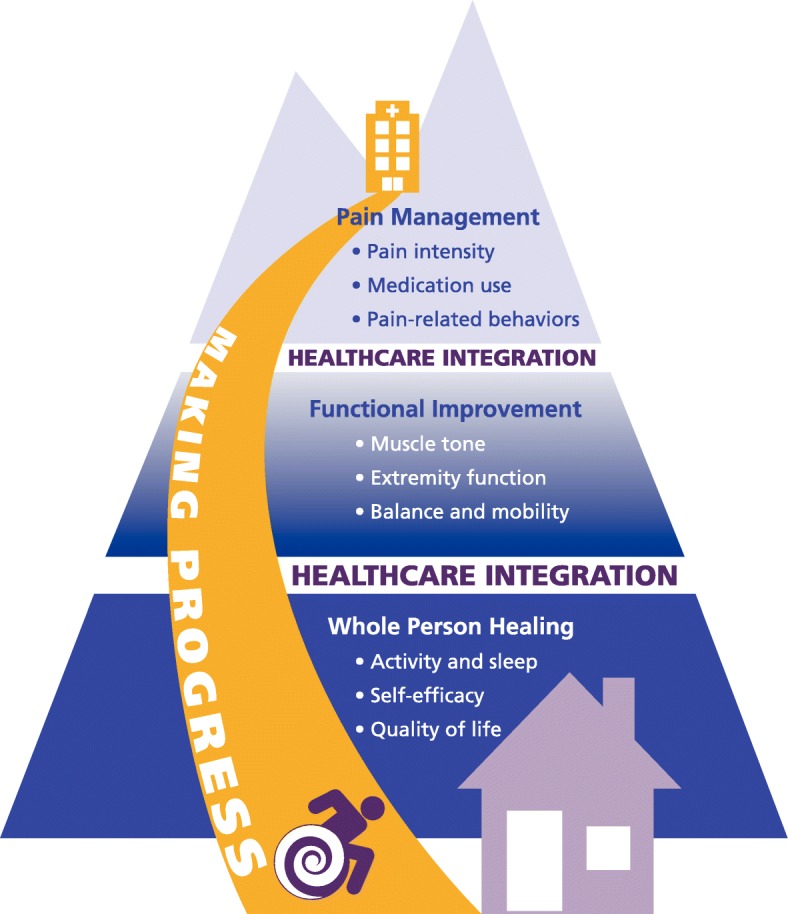
Stakeholder expectations from the integration of chiropractic care into a rehabilitation setting

Interviews were conducted with 60 participants, including 6 patients, 4 family members, 2 community members, and 48 facility staff. Our analysis produced a conceptual model of stakeholder expectations for chiropractic care (Fig. 1) with the central domain of *Making Progress.* In contrast to typical depiction of progress as a successful climb up a steep hillside, these patients made progress as an incremental journey down from the mountaintop rehabilitation hospital to home or other community setting, as depicted by the yellow road. The first domain stakeholders expected (or hoped) chiropractic care would address was *Pain Management*, an important hurdle for patients, though not the primary focus of their rehabilitation process. Next, stakeholders anticipated the DC would work with the therapy team to assist patients in gaining *Functional Improvement*, the main goal of their hospital stay. Collectively and throughout their admission, families and healthcare personnel also empowered patients to achieve *Whole Person Healing*. An additional domain, *Healthcare Integration*, illustrated by the white bands between themes, described how stakeholders anticipated the inclusion of chiropractic services would impact the broader delivery of healthcare services within this setting. Representative quotes in the text are identified by participant role (patient, family, nursing, etc.) and data type (individual interview, focus group).

### Making Progress domain

*“There’s a driving force on the mountain, and that is to make a positive impact on this person’s life as they try to recover from something that is very hard and a long journey. We know the journey doesn’t end here.”* (Nursing leadership focus group).

The overarching goal at this specialty hospital was to rehabilitate patients to allow discharge to the home or another community setting where the recovery journey would continue. For both staff and patients, a timely discharge was preferred.

*“We’re trying to get the patient as independent as possible as quickly as possible so that they can be discharged.”* (Therapy staff focus group).

*“I just as soon be home than here.”* (Patient interview).

Across stakeholder groups, the central domain of *making progress*, was described as an incremental process for patients with expected changes in both clinical outcomes and in how one views him- or herself as a person in the world.

*“The progress from when* [patients] *first come in to when they discharge, seeing what that progress is and how they’ve got better. How they’ve made their compensation for their new reality…It’s quite rewarding…watching patients come in, in various stages of post-acute* [injury], *and then see them, some of them, walk out of here. Even if they’re being wheeled out of here, they’re a completely different person and ready to take on the next challenges of life, which is pretty exciting.”* (Nursing leadership focus group).

### Pain management domain

*Pain management,* or the prevention and treatment of painful conditions, was a salient concern for all stakeholder goups. Rehabilitation patients often report the widespread occurence of both acute and chronic pain [23], as was noted for inpatients in this facility.

*“Most if not all our patients have some kind of pain they’re dealing with somewhere in their body. I probably can’t even think of one that said they weren’t having any type of pain due to the brain injury, spinal cord or stroke or whatever the injury was…”* (Therapy staff focus group).

Unmanaged pain was viewed as an impediment to the rehabilitation process and a reason some patients did not participate in their daily therapies and self-care activities.

*“I have pain to my left hip, ongoing, continual pain, and my shoulder is a lot better now for some reason, because I am not doing as much.”* (Patient interview).

*“*[My son] *is very healthy right now. But he has lots of pain. He’s got a lot of tone, a lot of spasticity. As soon as you touch his legs or try to stretch him, he yells and screams. When we go to move him to, say, do personal care, he yells and screams. He’s having a hard time with pain.”* (Family member interview).

The integration of chiropractic care into this setting was expected, or at least hoped, to bring about a direct effect on three aspects of pain management, including *pain intensity*, *medication use*, and *pain-related behaviors*. As a member of the therapy department reflected on how the addition of chiropractic care to the rehabilitation setting might aid patients:

*“If it’s* [chiropractic] *something that helps with their pain, I think it’s going to be a tremendous inclusion.”* (Therapy staff focus group).

#### Pain intensity

Most stakeholder groups thought chiropractic care could impact patients’ *pain intensity*, or the subjective experience of a graded amount of pain.

*“If they have ails and pains and bothersome things that the chiropractor feels he can help them with, then by all means.”* (Patient interview).

Participants with personal experience receiving spinal manipulation considered chiropractic adjustments as a means to bring immediate relief in pain intensity.

*“If a patient is having any pain… just a simple adjustment can make a difference in chronic pain or in discomfort.”* (Family member interview).

#### Medication use

Rehabilitation patients hoped that complementary treatments, such as chiropractic care, might allow them to take fewer medications:

*“I would consider anything to help improve my quality of life and lessen my reliance on painkillers, which I don’t really care to take.”* (Patient interview).

Medical and nursing staff discussed medication management more frequently than other stakeholders, and expected chiropractic care to lead to reduced use of pain medications.

*“Chiropractic care is not about the medicine and that’s a key point…that’s one less pill they would have to take getting rid of their pain.”* (Nursing staff focus group).

*“Minimizing the use of opioids and the other meds we use for pain would go a long way because all of those meds have side effects that hurt in other ways and that’s always a tough balance from the medication perspective.”* (Medical staff interview).

#### Pain-related behaviors

Pain-related behavior, including both cognitive changes and emotional responses, were expected to improve after the patient had a reduction in their overall pain. Nursing staff emphasized this expectation more than other stakeholders.

*“When you have brain injured* [patients] *that have pain, you get a lot of behavior from pain. It can be violent, it can be verbal, it can be all different sorts. When you have someone who is going through pain all the time and you have these behaviors a lot, that is hard to maintain every single day.”* (Nursing staff focus group).

“*Ultimately* [pain management] *allows them* [patients] *to be clear in their cognition. It allows them to be more independent because they’re safer, because they’re not altered from being on narcotics, so they can be more independent, more mobile. I imagine that would have an overall impact on the positive outcomes.”* (Nursing leadership focus group).

### Functional improvement domain

Stakeholders across groups shared the expectation that chiropractic might offer an additive impact on the *functional improvements* gained by patients in their daily work with therapy staff. Functional improvements were expected (or hoped) for in *muscle tone, extremity function*, and *balance and mobility*, all of which supported the patients’ ability to live in community settings.

#### Muscle tone

*Muscle tone and spasticity* were issues for many rehabilitation patients. Patient injuries often led to spasticity or laxity in the muscles, which, as described under pain management, was an uncomfortable experience. This concept of ‘tone’ was a common theme mentioned across many stakeholder groups, but emphasized by therapy staff.

*“The first thing that comes to mind for me is the muscle tone and the positioning, it being so imbalanced.”* (Therapy leadership focus group).

Since chiropractic care predominantly targets the neuromusculoskeletal system [20], some stakeholders thought these treatments may help manage muscle tone and contractures or decrease muscle spasms.

*“Anybody that has any problem with the back, even, you know, a muscle that is swollen,* [chiropractic] *relieves this.”* (Community member interview).

*“Someone with muscle spasms in their core could be benefited by adjusting some thing in their back or in their hips.”* (Nursing leadership focus group).

However, some stakeholders, particularly members of the therapy department, questioned whether chiropractic adjustments or joint manipulations would be beneficial for persons with muscle tone issues, particularly in persons with spinal cord injury.

*“That’s the big ethical question I have, too. If we’re doing it to a patient who is a quadriplegic or a paraplegic, my knowledge of adjustments and mobilizations and manipulations, is then to work on the muscles being able to hold those joints back into position. If somebody has absolutely no muscle tone…?”* (Therapy staff focus group).

*“I think it* [patient response to chiropractic] *will be mixed. I think some people will benefit from it and others might be more difficult because of high tone or they have hemiparesis, and that’s going to set things off constantly.”* (Therapy staff focus group).

#### Extremity function

Often interrelated to the issue of tone as a factor inhibiting mobility, an important goal for patients was gaining *upper and lower extremity function*, including greater *range of motion* in the joints. Improvements in how patients moved arms and legs was viewed as a marker toward gaining independence, and building self-efficacy. Though recovery in this area was, again, expected to be slow and incremental, chiropractic treatment was expected (or hoped) to help.

“[Chiropractic care] *would be a great help to me to get my arm and leg moving more.”* (Patient interview).

*“I’m thinking now specifically of shoulder subluxation, that’s just one joint that’s impacted by that change in tone because of the brain injury or stroke. How that would integrate in with what chiropractors are able to do. If you can fix my* [patient’s] *shoulder subluxation, tell me, come on board.”* (Therapy staff focus group).

Some patients, like those with certain spinal cord injuries, had conditions with more limited recoveries. In these cases, stakeholders offered tempered hopes for improvement in extremity function, but expected chiropractic care to help maintain range of motion and flexibility

*“We’d like to maintain the strength and flexibility that he does have, and keep him as limber as possible.”* (Family member interview).

*“Be limbered up a little bit with exercise and different movements.”* (Patient interview).

#### Balance and mobility

Some participants thought chiropractic care, in concert with therapy treatments, might improve *balance and mobilty*.

“*I’d like to be able to walk with a cane but I can’t. I don’t have the balance.*” (Patient interview).

*“He’ll walk like this* [demonstrates a side-tilted posture] *if he gets tired. Maybe that* [chiropractic care] *would help him to have more of an upright posture if his muscles weren’t tight*.”(Nursing staff focus group).

Physical comfort while using a wheelchair were expected to improve with chiropractic care.

“*Spending a day in a wheelchair is not comfortable. Being just a little bit out of alignment can get exaggerated over just an hour*.”(Therapy staff focus group).

Other stakeholders expected chiropractic care to aid in the patients’ ability to transfer from a bed to a wheelchair and to position themselves with less assistance from staff.

“*Mobility, like patients that are limited for reasons of pain or muscle spasm and tone. Independence and bed mobility, and even the simplest thing as turning themselves on their side. They’re going to have help, increased independence with that* [from chiropractic care].”(Nurse leadership focus group).

### Whole person healing domain

The goals of pain management and restoration of physical function were focused on the curative aspect of care delivery, returning the patient to a state of health that was similar to where they were in the past. Building upon the curative goals, all stakeholder groups mentioned a higher-order goal of *whole person healing* for rehabilitation patients. Whole person healing is the acceptance of change and a new sense of self which may mean functioning differently or in a different capacity than previously experienced [24]. This acceptance of change is required to successfully transition patients to a readiness for discharge and self-care. Both staff and family members were supportive of the patient achieving whole person healing goals, including improving *activities of daily living (ADLs) and sleep*, mastering a sense of *self-efficacy*, and finding an acceptable *quality of life*.

#### Activities of daily living and sleep

Stakeholders described chiropractic care as a means of improving body positioning during the completion of ADLs. Sleep, especially, was thought to be more tolerable after a chirpractic adjustment.

“[Chiropractic care] *helps them in their daily life, getting them sitting upright whether it’s swallowing and breathing, eating, all those things, accessing their environment*” (Therapy staff focus group).

*“Improved sleep. More relaxed. If your spine’s out of whack, it affects everything you do, including sleep, including your ability to just take care*.” (Nurse leadership focus group).

*“Participation across their schedule. Daily engagement in anything from getting out of bed in the morning to attending meals, therapy.”* (Therapy staff focus group).

#### Self-efficacy

Some participants viewed the DC as a clinician who might support rehabilitation patients in gaining confidence and self-efficacy in their ability to care for themselves.

“*It* [chiropractic program] *will also be somewhat empowering for individuals where…* [the] *individual has to take charge of their recovery*.” (Administrative staff interview).

“*A person can attain whatever they want to attain. This is building the self-confidence in individuals.”* (Community member interview).

#### Quality of life

Along with family and other staff, the chiropractor was considered a healthcare professional who might contribute to the overall quality of life of the rehabilitation patient.

*“Success in our patient population is improved quality of life, improved independence, ability to go to the next environment in a better place…the successful integration of a chiropractor would be that they have something to offer the patient that would help them on that pathway.”* (Medical staff interview).

*“Increase their* [the patient’s] *quality of life while they’re here.”* (Patient interview).

### Healthcare integration domain

The collaborative and integrative culture of this rehabilitation hospital led stakeholders to share expectations extending beyond that of a direct impact from chiropractic care on the patient’s condition. Chiropractic care also was anticipated to facilitate patient progress by its indirect effect on the work of the broader interdisciplinary team.

*“The team, how we all work together and support each other, talk about what’s going on and try best to help the client through whatever challenge they have…looking at all aspects of their healing, too, bringing in everybody. Not just ‘we’re working well as a team’, but also what aspects of the client are going to best help them to heal.”* (Therapy staff focus group).

Chiropractic was hoped to increase patient engagement in other treatment modalities.

*“For some people, pain affects their therapy and when your pain affects your therapy, that affects your ability to move on. I’d like to think that people’s transformation and ability to get better would be faster and easier*.” (Nursing staff focus group).

*“When that* [muscle tone] *becomes loosened up, it might help with other things to flow more freely and to work more easily with physical therapy.”* (Family member interview).

Several stakeholders expected adequate pain management to prompt a cascade effect in other patient outcomes. That is, once pain was managed, a patient might achieve exponential gains toward functional and healing goals. Chiropractic care was considered, by some participants, as a means of stimulating such a cascade effect.

*“If you bring in chiropractic and improve their lives you can get them off the medication and back into functioning, in a job, doing something meaningful for society as far as their own wellbeing and their own care.”* (Nursing staff focus group).

Some staff also anticipated that the addition of chiropractic services would impact the broader concerns of the healthcare organization, such as through shorter lengths of stays.

*“As far as therapeutically, patient stays getting shorter because maybe* [chiropractic is] *relieving* [patient’s] *pain so they’re participating more and therefore making better progress.”* (Therapy staff focus group).

However, participants with less familiarity, either personally or professionally, with chiropractic care were skeptical about its potential impacts.

*“I don’t know what the indications are for chiropractic care, but…if it can be helpful, I’m a practical person. If it works, then I’m happy to use it and try it and have people do whatever they do that helps people. It sounds like chiropractic stuff’s good for back pain and other pain issues. I don’t know how it translates to other pain phenomenon in general, but if it works, then I think it’s a great thing. I think the people out there want other things than meds.”* (Medical staff interview).

## Discussion

This qualitative study offers new insights into stakeholder expectations from the integration of chiropractic services into inpatient healthcare facilities, such as the rehabilitation specialty hospital that served as the setting for this research. Despite this being a unique setting for the addition of chiropractic care, research on chiropractic integration into different hospital settings in Canada and the US has demonstrated very similar themes [16, 25]. Patients, families, and providers affirmed the notion that the chiropractor’s overall contribution to the healthcare team was to support rehabilitation patients in their quests of *making progress* toward discharge to the community. Within this framework, stakeholders expected that the chiropractors’ primary focus would be on *pain management*, with clinically demonstrative *functional improvements* another likely outcome. Other work has demonstrated the value of chiropractic diagnosis of musculoskeletal conditions in integrated settings [26, 27], however participants in this rehabilitation setting focused on expectations of outcomes related to treatments. Studies in both chiropractic clinics and multidisciplinary healthcare settings have identified pain and functional disability as expected outcomes of chiropractic care [13–15, 28]. These measures also are those most commonly used to evaluate the outcomes of spinal manipulation in randomized clinical trials [28, 29]. *Whole person healing* is not well described as an outcome of chiropractic care, although previous works have identified its components, such as quality of life and self-efficacy, as potential therapeutic elements [9, 24, 30, 31]. The final domain, that of *healthcare integration*, also is not broadly discussed for chiropractic care.

Treatment expectancies of patients using complementary therapies, including chiropractic care, for chronic LBP have identified four domains for improvement: pain relief, improved function and ability to engage in meaningful activities, physical fitness, and overall well-being including mental health [9]. Our findings echo these domains, but with more nuanced meanings for the rehabilitation patient, his or her family, and professional caregivers. This difference in nuance may be expected as neurorehabilitation patients often have complex health conditions beyond those experienced by persons with chronic LBP [17]. For example, expectations in our study extended beyond pain relief [9] to address facets of long-term pain management, including the ongoing use of pain medications and the impact of chronic pain on pain-related behaviors.

Hsu et al. also incorporated concepts such as muscle strength and flexibility under the domain of physical fitness, in which the back pain sufferer might achieve increased physical capacity within an ambulatory body, and function under the ability to engage in activities of daily living, work, and social interactions [9]. In our study, patients were recovering from a traumatic brain injury, spinal cord injury, or cerebrovascular accident; many individuals will use a wheelchair or other mobility aids for the remainder of their lives. Thus, the functional improvements these patients expected were of a modest scope, but more difficult to achieve, such as being able to assume prolong sitting postures in a wheelchair or turning over in bed unassisted. Little is known about the impact of chiropractic care in persons who rely upon other people for movement assistance or for who rely upon wheelchairs for mobility [17, 18].

Many participants emphasized an expectation that chiropractic care might affect “tone” in the rehabilitation patient population. Tone was not well-defined by participants, even when encouraged to elaborate on the concept during interview sessions. And yet, tone was viewed as a major barrier to patient progress, especially among therapy staff and family members, many of whom reported hearing about the concept of tone from therapy staff. Patients with spinal cord injuries often experience muscle flaccidity followed by the development of hypertonicity and spasticity, increasing over time, though the reasons behind this phenomenon remain uncertain [32]. These physiological qualities may be equivalent to the idea of tone discussed by participants in this study. Alternatively, tone may be viewed as a vitalistic concept beyond a strictly musculoskeletal application, acting as a surrogate for a restoration of balance, homeostasis, or neurological tension and a return to overall health [33]. In this context, a desired treatment effect on tone from chiropractic care may represent a hope to meet the shortcomings of treatment modalities already provided. To our knowledge, clinical research has not evaluated sufficiently the effect of chiropractic care on tone using either definition, or how tone might differ from other concepts in the chiropractic literature, such as muscle stiffness [34]. Given the emphasis of this topic, further investigation into both the physical and philosophical aspects of tone as it relates to patient recovery seems warranted.

While no participant described a scenario in which chiropractic care resolved (that is, cured) the neurological damage to the brain or spinal cord sustained by these patients, several believed that spinal manipulation might offer unique benefits to patients over current therapies. This finding falls short of Jamison’s case study of 146 patients in 6 Australian chiropractic clinics which reported that one-third of participants attributed their clinical benefits solely to the chiropractic adjustment, while 85% believed the adjustment contributed to over half of their positive outcome [35]. However, for some patients, families, and clinical staff in our study, the expected outcomes of chiropractic care were high, which may not be achievable in this patient population. Medical and chiropractic providers may attempt to realign patient expectations with what they consider reasonable to increase patient satisfaction with care [36]. Further, expectations can change over time, making this topic an ongoing process of patient-provider communication [36].

Unexpectedly, some participants described a hoped for ‘cascade effect’ from chiropractic care in which improved pain management would increase patient engagement in other therapeutic modalities, such as physical and occupational therapy, and that together these combined treatments could lead to decreased functional limitations and better patient focus on his or her longer-term goals. This desired cascade effect among clinical outcomes is not dissimiliar to the interrelationships of domains for LBP treatments described by Hsu and colleagues [9]. The evolution of expectations by and for these rehabilitation patients from pain relief to a long-term focus on whole person healing is similar to changes in expectations reported by patients receiving 4 complementary therapies, including chiropractic [11]. However, clinical research in chiropractic has not sufficiently explored such a cascade effect in patient centered outcomes, which may be a topic for future exploration.

Prior work on the inclusion of nurse practitioners into Canadian healthcare systems identified intention as a central concept in the process of integration, which may define how the role of the practitioner will fit within the context of the healthcare team and which patients are suitable for the role of that practitioner [37]. The intentions of other stakeholders may inform the chiropractor not only of an expected team role, such as during orientation to the new position, but may serve as an evaluation framework to gauge the success of the integration process [38]. In the current study, stakeholders had the expectation that a DC would focus on pain management, and collaborate with members of the therapy staff on functional outcomes. Few expectations for collaboration between a DC and other departments, such as medicine and nursing, were described. A prudent approach for chiropractors integrating into new clinical settings would be to help set clear expectations for areas of clinical focus that are based on sound scientific evidence, such as pain relief, rather than a desire to please new colleagues and patients by promising outcomes in other areas [15].

### Limitations

The purposive sample for this study were volunteers from a rehabilitation hospital who may have had more positive views of chiropractic and its integration than similar stakeholders in other multidisciplinary settings, such as cancer care settings, veterans hospitals, or ambulatory care clinics. Several studies note that recent or current users are more likely to endorse chiropractic than those who have not received such care [39, 40]. Patients with neurological conditions that limited their communication abilities were not able to participate in these interviews, potentially reducing the generalizability of expectations to others with or without similar communication limitations [19].

Stakeholders often did not differentiate between their expectations and their hopes for the anticipated chiropractic program. Expectations are thought to reflect likely outcomes whereas hopes represent optimal outcomes [9, 36, 41, 42]. This distinction may not be clear to stakeholders or newly integrating chiropractic providers in similar settings. Further, stakeholders were asked about their expectations related to integrating a chiropractor into this rehabilitation hospital. They were not asked about expectations of clinical outcomes explicitly, which could have influenced responses.

Due to logistical constraints, we were limited in our ability to conduct member checks to ascertain whether our interpretation of participants’ expectations was in line with their actual viewpoints. Interview sessions often ended with an overview of keypoints, which to some extent allowed for additional discussion of participant meaning. We are encouraged that our results do reflect the general perceptions of the patients, families, and staff of CMSH given that many of the expectations we identified in this analysis were mentioned across individuals and by multiple members of the focus groups. To further add to the credibility of our results, our team engaged in multiple coding rounds, had findings reviewed by investigators who were employees of the rehabilitation hospital, and offered extended quotes for the reader to use to judge the transferability of our findings to other contexts in which chiropractic care is delivered [19, 43].

## Conclusion

This qualitative study described stakeholder expectations of chiropractic care in a neurorehabilitation context, which expands the growing body of knowledge on the integration of chiropractors into multidisciplinary settings. We found consistent expectations for chiropractors to engage in pain management with rehabilitation patients, and to collaborate with therapy providers on functional improvements in this population. Making progress toward discharge, whole person healing, and healthcare integration were novel domains where chiropractors might also contribute to patient and facility outcomes. Some expected outcomes of chiropractic care, such as improvement of muscle tone, are not well-supported in the literature, and may lead to patient and provider dissatisfaction with chiropractic care. Our results suggest an opportunity to explore an expanded array of patient-reported outcomes in chiropractic clinical research.

## Abbreviations

ADLs: Activities of daily living
CAM: Complementary and alternative medicine
CMSH: Crotched Mountain Specialty Hospital
DC: Doctor of chiropractic
LBP: Low back pain
MSK: Musculoskeletal
